# Incidence and Timing of Immune-Related Adverse Events in Immune-Checkpoint Inhibitor-Treated Patients: A Retrospective Observational Study

**DOI:** 10.3390/jcm12247564

**Published:** 2023-12-08

**Authors:** Kou Masaki, Motoyasu Miyazaki, Hideki Kakimoto, Yuma Fukiage, Haruka Fukue, Akio Nakashima, Osamu Imakyure

**Affiliations:** 1 Department of Pharmacy, Fukuoka University Chikushi Hospital, Chikushino 818-8502, Japan; masakikou@fukuoka-u.ac.jp (K.M.); kakimoto0917@fukuoka-u.ac.jp (H.K.); fukiage@fukuoka-u.ac.jp (Y.F.); fukue@fukuoka-u.ac.jp (H.F.); anakashima@fukuoka-u.ac.jp (A.N.); imakyure@fukuoka-u.ac.jp (O.I.); 2 Faculty of Pharmaceutical Sciences, Fukuoka University, Fukuoka 814-0180, Japan

**Keywords:** immune-related adverse events, immune-checkpoint inhibitor, observational study

## Abstract

Background: Immune-checkpoint inhibitors (ICIs) are effective against various cancers; however, immune-related adverse events (irAEs) have been reported and the timing and risk factors are unknown. Therefore, we examined the incidence and timing of irAE occurrence. Methods: Patients who received ICIs at our hospital between 1 April 2016 and 31 March 2020 were enrolled. Patients were classified into an irAE group or non-irAE group. In addition, we examined the onset time and symptoms of irAEs for each ICI type. Results: A total of 80 patients received ICIs, of which 27 (33.8%) developed irAEs. The incidence of irAEs was 35.3% for nivolumab, 35.5% for pembrolizumab, and 28.6% for atezolizumab. The incidence of pneumonitis was 12.5%, 8.8% for dermatologic adverse events, and 6.3% for thyroid dysfunction. The earliest case of onset was after the 1st course, and the latest cases occurred after the 66th course. By the sixth course, 69% of the irAEs occurred. The positive rates for anti-thyroid peroxidase and anti-thyroglobulin antibodies were higher in the irAE group compared to the non-irAE group. Conclusions: Our findings suggest a high probability of irAEs occurring early in ICI treatment, with a diverse range of symptoms. This underscores the need for vigilant monitoring and tailored patient management during the initial courses of ICI therapy.

## 1. Introduction

In recent years, immune-checkpoint inhibitors (ICIs), such as anti-PD-1 antibodies (nivolumab and pembrolizumab), anti-PD-L1 antibodies (atezolizumab and durvalumab), and anti-CTLA-4 antibody (ipilimumab), have demonstrated remarkable efficacy for a variety of solid tumors and hematologic malignancies, resulting in the expansion of their clinical indications. Furthermore, the combined use of ICIs as well as their synergy with cytotoxic chemotherapeutic drugs, small molecular agents, anti-angiogenic drugs, and radiation therapy, has improved therapeutic outcomes [1,2,3]. ICIs activate T cells by inhibiting immune-checkpoint molecules that suppress the immune response, resulting in enhanced immunity [4]; however, the onset mechanism and occurrence of side effects are different from those of conventional cytotoxic drugs and molecular targeted agents.

Immune-related adverse events (irAEs) that cause autoimmune-disease-like symptoms occur because of a breakdown in the immune response. Such irAEs cause damage to the skin, lungs, liver, large intestine, nerves, and endocrine system, and their unpredictability in terms of onset and severity adds a layer of complexity to ICI therapy [1,2,3]. Although there have been some reports describing the timing of the onset and risk factors for irAEs, they remain unclear. There are no regularities in the symptoms and timing of their onset, and it is difficult to predict them in advance. As a result, there are many cases in which treatment must be discontinued [2,5].

At Fukuoka University Chikushi Hospital, instances of irAEs following ICI administration were documented. Although the incidence of irAEs for each specific ICI have been reported [2,3], there are few reports examining the incidence of irAEs among drugs, symptoms at onset, and timing. The aim of this study was to determine the incidence rate and timing of irAEs among various ICIs. Our findings suggest a high probability of irAEs occurring early in ICI treatment, with a diverse range of symptoms. Not only the incidence, timing, and symptoms of irAE but also the treatment for irAE and subsequent cancer treatment were summarized in this study, which may help in treatment strategies in actual clinical practice.

## 2. Materials and Methods

### 2.1. Study Patients

**Figure 1:**
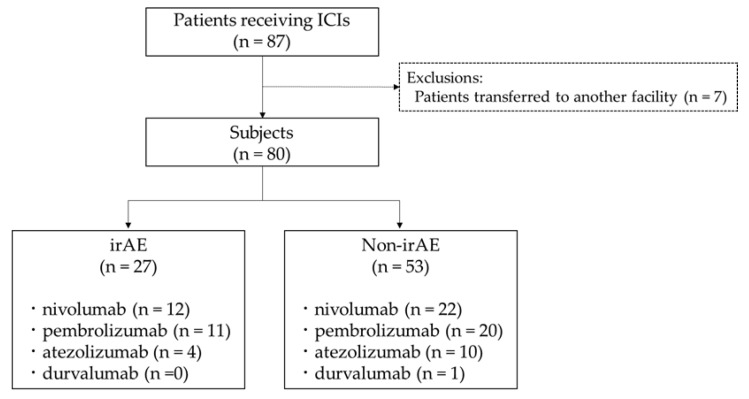
The study flowchart.

The study included patients who received ICIs, specifically nivolumab, pembrolizumab, atezolizumab, and durvalumab, at our hospital over a five-year period from April 2016 to March 2020. Patients who were administered ICI therapy but were transferred to another facility were excluded (Figure 1). The observation period extended until March 2022. The Ethics Committee of the Fukuoka University School of Medicine approved the study protocol (No. C23-06-003). Patient consent was waived due to the retrospective design of the study. We used the opt-out method. The disclosure document for this clinical study was provided by the Fukuoka University website.

### 2.2. Clinical Characteristics

Patient data for the following variables were extracted from the electronic medical records: age; gender; body mass index (BMI); cancer type; treatment regimen; number of treatment cycles; reasons for treatment discontinuation; history of interstitial lung disease (ILD), chronic obstructive pulmonary disease (COPD), and autoimmune disease; concurrent use of proton pump inhibitors (PPIs); status of anti-thyroid peroxidase (anti-TPO) and anti-thyroglobulin (ant-Tg) antibodies; and various biochemical parameters, including white blood cell (WBC), red blood cell (RBC), hemoglobin (Hg), platelet (PLT), albumin (Alb), creatinine (Cr), creatinine clearance (CCr), estimated glomerular filtration rate (eGFR), total bilirubin (T-Bil), aspartate aminotransferase (AST), and alanine aminotransferase (ALT). CCr was calculated using the Cockcroft–Gault formula.

### 2.3. Definitions

During the observation period, patients who developed irAEs were classified as the irAE group, whereas those who did not were categorized as the non-irAE group. Patients who died during treatment and patients whose ICI treatment was terminated because of decreased performance status or progressive disease were placed into the non-irAE group. We investigated the grading of irAEs assessed by Common Terminology Criteria for Adverse Events (CTCAE) version 5.0. Clinical characteristics were compared between the irAE and non-irAE groups. For the irAE group, the timing, manifestations of irAEs, treatment for irAEs, and subsequent cancer chemotherapy were analyzed for each specific ICI.

### 2.4. Statistical Analysis

Statistical analysis involved comparing the irAE and non-irAE groups using a chi-squared test or Fisher’s exact test for gender, anti-TPO and anti-Tg antibodies, history of interstitial lung disease, and concurrent PPI use. For continuous variables, such as age, BMI, WBC, RBC, Hb, PLT, Alb, CCr, eGFR, T-Bil, AST, and ALT, the Wilcoxon rank-sum test was used. Continuous variables were reported as the median (interquartile range (IQR)), and *p*-values less than 0.05 were considered statistically significant. The analysis was performed using JMP^®^, version 12.0.1 software (SAS Institute, Tokyo, Japan).

## 3. Results

### 3.1. Incidence of irAEs

Over a 5-year study period, a total of 87 patients received ICIs at our hospital, and 80 met the inclusion criteria (Figure 1). The distribution of ICIs included 34 patients on nivolumab, 31 on pembrolizumab, 14 on atezolizumab, and 1 on durvalumab. Of these, 27 patients (33.8%) developed irAEs. The incidence rates for each drug were as follows: 35.3% for nivolumab (12 of 34), 35.5% for pembrolizumab (11 of 31), 28.6% for atezolizumab (4 of 14), and 0% for durvalumab (0 of 1). Of note, among the eleven patients who developed irAEs with pembrolizumab, two first experienced thyroid dysfunction before subsequently developing pneumonitis.

### 3.2. Patterns of irAE Manifestation

**Figure 2:**
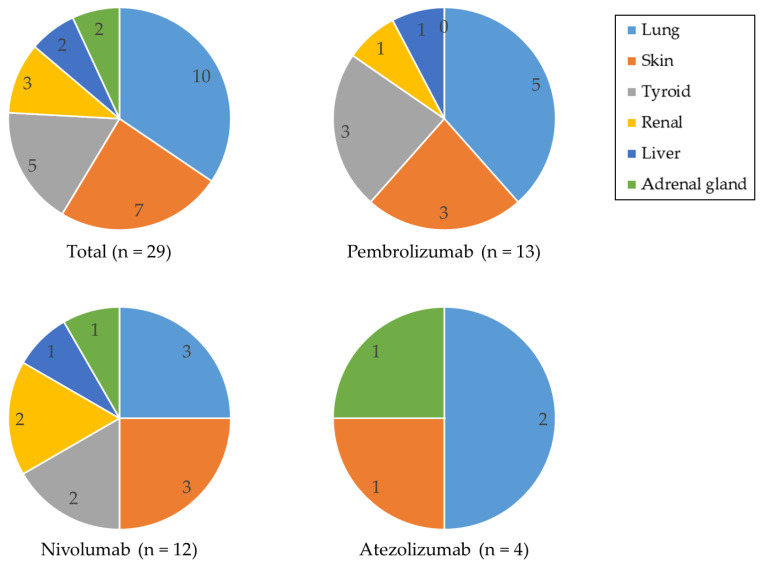
The classification of symptoms for each specific ICI.

Of the twenty-seven patients who developed irAEs, two first experienced thyroid dysfunction and later developed pneumonitis, resulting in a total of 29 irAE cases. Based on irAE symptoms, there were ten pneumonitis, five thyroid dysfunction, seven dermatologic adverse events, two hepatitis, three acute kidney injuries, and two adrenal insufficiency cases (Figure 2). An analysis of irAE patterns revealed diverse manifestations, with pneumonitis being the most common, followed by dermatologic events and thyroid dysfunction.

Among all ICIs, pneumonitis cases exhibited the highest incidence at 12.5% (10 of 80), followed by dermatologic adverse event at 8.8% (7 of 80) and thyroid dysfunction at 6.3% (5 of 80). When analyzed by individual ICI, nivolumab and pembrolizumab exhibited similar patterns to the overall ICI group. For pembrolizumab, however, the incidence rates for dermatologic adverse event and thyroid dysfunction were both 9.7% (3 of 31). For nivolumab, the incidence of pneumonitis was slightly lower at 8.8% (3 of 34) compared to the entire ICI group. Additionally, thyroid dysfunction and acute kidney injury rates were both 5.9% (2 of 34). In contrast, atezolizumab did not induce thyroid dysfunction but had a 7.1% incidence of adrenal insufficiency (1 of 14).

### 3.3. Timing of irAEs

**Figure 3:**
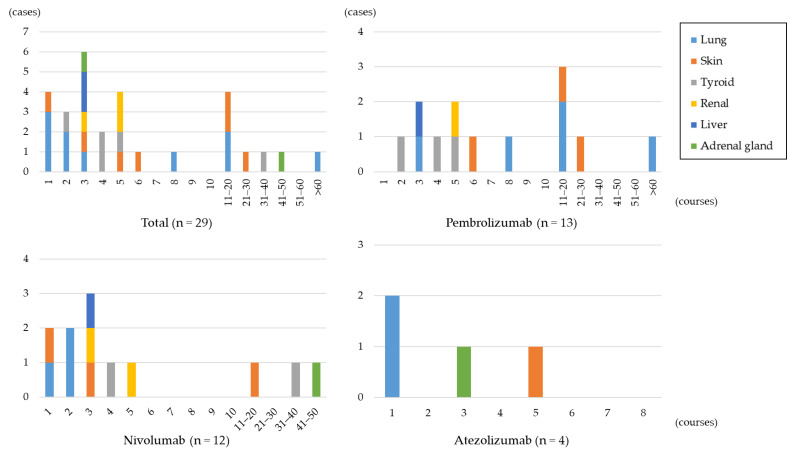
The number of irAE cases and the timing of onset for each specific ICI.

Figure 3 shows the number of irAE cases and the timing of their onset for each ICI type. The earliest onset was observed with nivolumab and atezolizumab, which occurred after the first course. The latest onset occurred with pembrolizumab, with one case occurring after the 66th course. Up to the sixth course, 69% of all irAE cases (20 of 29) had manifested. Acute kidney injury (3 of 3) and hepatitis (2 of 2) emerged by the fifth course. Among the five cases of thyroid dysfunction, four had developed by the fifth course. Of the ten pneumonitis, six had manifested by the third course, with sporadic occurrences thereafter. The latest pneumonitis case emerged after the 66th course, suggesting a tendency for early onset but ongoing incidences throughout the entire observation period. Similarly, dermatologic adverse events followed a similar pattern, with four of seven cases occurring within the first six courses and sporadic onsets occurring up to the 27th course. Among the two adrenal insufficiencies, one emerged after the 3rd course and the other after the 47th course.

Among the twelve patients who developed irAEs from nivolumab, nine (75%) had an onset within the first five courses. Additionally, three cases involved the use of ipilimumab, an anti-CTLA-4 antibody. One patient experienced pneumonitis after the second course, one had hepatitis after the third course, and one developed thyroid dysfunction after the fourth course. Among the thirteen cases developed irAEs with pembrolizumab, seven (53.8%) had an onset within the first six courses. All three cases of thyroid dysfunction occurred within the first five courses. Dermatologic adverse events had an onset between the 6th and 27th courses and pneumonitis between the 3rd and 66th courses. All four cases of irAEs associated with atezolizumab manifested within the first five courses. Two cases of pneumonitis occurred after the first course, although both patients had a history of interstitial lung disease.

In the irAE group, four cases involved concurrent chemotherapy. Two of these were from pembrolizumab, with one developing dermatologic adverse event after the 27th course. The other patient experienced multiple irAEs, with thyroid dysfunction after the fourth course, followed by pneumonitis after the eighth course. In the two patients treated with atezolizumab, one developed adrenal insufficiency after the third course, and the other had a history of interstitial lung disease, developing pneumonitis after the first course.

### 3.4. Clinical Characteristics between irAE Group and Non-irAE Group

Table 1 shows the clinical characteristics between the irAE group and non-irAE group. The rates of positive anti-TPO (48.2% vs. 17.0%, *p* = 0.003) and anti-Tg (40.7% vs. 17.0%, *p* = 0.020) antibodies, which are considered useful for predicting thyroid dysfunction [6], were significantly higher in the irAE group compared with the non-irAE group. All five patients with thyroid dysfunction were positive for the anti-TPO and anti-Tg antibodies. The rate of concomitant use of ipilimumab was significantly higher in the irAE group compared with the non-irAE group (14.8% vs. 0%, *p* = 0.011). No significant differences were observed in other clinical characteristics, including a history of ILD, COPD, or autoimmune disease, between the two groups.

### 3.5. Grade of irAEs, Treatment for irAEs, and Subsequent Cancer Therapy for irAE Group

The grade of irAEs, treatment for irAEs, and subsequent cancer chemotherapy for the 29 cases that developed irAEs are shown in Table 2. Of the 29 cases who developed irAEs, 7 had grade 3 adverse events, and 22 had grade 2 or lower. No cases developed grade 4 or 5 adverse events, but 10 cases required hospitalization. ICI treatment was temporarily discontinued due to irAEs in 19 of the 29 cases. In addition, 25 cases required systemic treatment such as steroids and thyroid hormone replacement, and symptomatic treatment such as topical steroid treatment. Of the 29 cases, 11 cases did not receive subsequent cancer therapy. One case (patient ID 13) was determined to have a progressive disease after the onset of irAE and thus was switched from nivolumab (anti-PD-1 antibody) to atezolizumab (anti-PD-L1 antibody).

## 4. Discussion

In the context of immune responses against cancer, the primary mechanism of antigen recognition and attack is orchestrated by CD8+ T cells. The activation of CD8+ T cells requires the recognition of the major histocompatibility complex as the primary stimulatory signal and co-stimulatory molecules (such as CD28) [7,8]. Concurrently, immunosuppression involves regulatory T cells and other factors that prevent excessive activation. These factors are collectively referred to as immune-checkpoint molecules, which include CTLA-4 and PD-1/PD-L1, and their implication in autoimmune diseases [9]. These checkpoint molecules can be targeted by antibodies known as ICI therapy; however, the phenomenon of irAEs emerges as a consequence that resembles autoimmune or inflammatory disorders, highlighting the disruption of normal immune regulation [10]. The timing of onset, risk factors, and mechanisms of irAEs resulting from ICI administration have not been fully elucidated. The early detection and treatment of irAEs are considered central to countermeasures, and if the trends in side effects for each drug are known, it will be possible to select drugs associated with a safer profile.

### 4.1. Incidence Rate and Timing of irAE

The data presented in this study are consistent with the varying rates of irAEs across different ICI treatments. Incidence rates of irAEs reported by Xu et al. for adverse events (grade 1–5) associated with nivolumab, pembrolizumab, and atezolizumab were 71.8%, 75.1%, and 66.4%, respectively [11]. In contrast, we found an overall irAE incidence rate of 33.8%, with 35.3% for nivolumab, 35.5% for pembrolizumab, and 28.6% for atezolizumab. This variance may be attributed to differences in the study population, patient background, and criteria for diagnosing irAEs. Although there have been various reports, the exact time of irAE onset has not yet been established. In a pooled analysis of 8436 patients from 23 clinical trials treated with ICIs, the median onset of irAEs was 2.2–14.8 weeks after administration [12]. In the present study, 65.5% (19 of 29 cases) of ICI-treated patients developed symptoms by the fifth course, which was not significantly different from previous reports.

### 4.2. Symptoms of irAEs

#### 4.2.1. Pneumonitis

There are various reports on an incidence rate of 2–10% in patients with anti-PD-1/PD-L1 antibody therapy alone [13,14]. Although anti-CTLA-4 antibodies are considered rare at less than 1%, the incidence increases with combination therapy [15,16]. In the present study, the incidence rate of pneumonitis was 12.5% (10 of 80 cases), which is slightly higher compared to previous reports [13,14]. Risk factors for the development of pneumonitis, such as interstitial pneumonia, include lung lesions, post lung surgery, decreased respiratory function, oxygen administration, and radiation exposure [17]. At our hospital, the incidence of pneumonitis cases with atezolizumab was 14.3% (2 of 14 patients), but the incidence of pneumonitis reported in the study by Socinski et al. was 2.3% [18]. The two cases of pneumonitis caused by atezolizumab in the present study resulted from a history of interstitial pneumonia, which is consistent with a previous report [17]. In the present study, pneumonitis occurred by the second course of nivolumab and occurred by the eleventh course of pembrolizumab in all but one patient. Pooled analysis by Si-Qi et al. reported that pneumonitis with PD-1/PD-L1 antibody alone occurred at 7.8–27.9 weeks [12], and the results in the present study were within the range of previous reports. The exact reason why the onset time of pneumonitis differs between different ICIs is unclear. Of the ten cases who developed pneumonitis in this study, there were two cases each with a history of interstitial pneumonia and COPD, and three out of these four cases developed pneumonitis after the first or second course. A history of respiratory disease, rather than drugs, may be associated with earlier onset of pneumonitis. Further investigation is required to clarify these relationships.

#### 4.2.2. Thyroid Dysfunction

Based on a systematic review, the incidence of hypothyroidism is 8.0–8.5% with anti-PD-1 antibody alone and 4.7–6.0% with anti-PD-L1 antibody alone, and the incidence of hyperthyroidism (toxicosis) is 2.8–3.7% with anti-PD-1 antibody alone and 2.3% with anti-PD-L1 antibody alone [19]. All of the thyroid dysfunctions that occurred in the present study were hypothyroidism, with an incidence rate of 5.9% (2 of 34 cases) for nivolumab and 9.7% (3 of 31 cases) for pembrolizumab. Compared to previous reports, the incidence rate was slightly lower for nivolumab and slightly higher for pembrolizumab, but the incidence rate as an anti-PD-1 antibody was 7.7% (5 of 65 cases). Furthermore, it was reported that the incidence of thyroid dysfunction is higher when anti-TPO and anti-Tg antibodies are positive [6]. In the present study, anti-TPO and anti-Tg antibodies were also detected in all cases with thyroid dysfunction. Some previous studies found that when pembrolizumab was used alone, the median time to onset was 6 weeks (IQR: 3–40 weeks) [20] and 3.5 months (IQR: 1 day to 18.9 months) [21]. With nivolumab alone, the median time to onset was 2.9 months (IQR: 1 day to 16.6 months) [21]. It can occur immediately after administration or in cases in which treatment was continued for longer than 1 year. In the present study, one case each occurred during the fourth and thirty-first courses of nivolumab, and three cases occurred during the second to fifth courses of pembrolizumab.

#### 4.2.3. Dermatologic Adverse Event

In clinical studies of nivolumab and pembrolizumab, when administered alone, dermatologic adverse event occurred in 30–40% of all grades. When administered in combination with anti-CTLA-4 antibodies, the incidence was 60–70% [22,23]. Conversely, it does not increase when combined with chemotherapy [24]. There is also a report that out of 70 patients treated with atezolizumab for renal cell carcinoma, approximately 20% developed a grade 1 dermatologic adverse event [25]. However, among the seven cases in the present study, there were no cases in which anti-CTLA-4 antibodies were used in combination. Chemotherapy was used in combination with pembrolizumab in only one case. Furthermore, when anti-PD-1 or anti-PD-L1 antibodies were administered alone, the incidence rate was 9% (6 of 67 cases), which was different from that of previous reports. This may be because there were cases in which a definitive diagnosis of skin-related irAE could not be reached because of mild grade 1 skin symptoms. Dermatologic adverse events often occur after the second course of administration, with reports of an average of 5 weeks with anti-PD-1 antibody alone and 2 weeks with the combination of anti-PD-1 and anti-CTLA-4 antibodies [26,27]. In the present study, the earliest onset was after the first course and the latest onset was after the twenty-seventh course; thus, it is possible that onset can occur during the entire period.

#### 4.2.4. Hepatitis

Hepatitis occurs in 1–17% of patients receiving ICIs [28]. The incidence rate in this study was 2.5% (2 of 80 cases), which was within the range of previous reports, thus supporting our results. The time until the first onset of hepatitis was 8–16 weeks with anti-PD-1/PD-L1 antibodies, 8–9 weeks with anti-CTLA-4 antibodies, and 6–9 weeks when used in combination [12]. In the present study, there was one case each of nivolumab and pembrolizumab, and the time of onset was during the third course. Thus, the time of onset was within the range reported in previous studies.

#### 4.2.5. Acute Kidney Injury

The incidence of acute kidney injury when nivolumab and pembrolizumab are administered alone is 1.9% and 1.4%, respectively, and 4.9% when combined with ipilimumab and nivolumab [29]. Alternatively, according to clinical data by Seethapathy et al., the incidence of persistent renal dysfunction was 10% with anti-CTLA-4 antibody alone, 7% with anti-PD-1 antibody alone, 8% with anti-PD-L1 antibody alone, and 7% with combination therapy [30]. In the present study, the rates of acute kidney injury were 5.9% (2 of 34 cases) with nivolumab, 3.2% (1 of 31 cases) with pembrolizumab, and 3.8% (3 of 80 cases) of all ICIs. Although there are reports that acute kidney injury is accompanied by irAEs in other organs [30], there were no cases in which other irAEs occurred concurrently in the present study. Additionally, there are various reports regarding the risk of developing acute kidney injury due to irAEs. PPI use, low baseline eGFR, and combination therapy with anti-CTLA-4 and anti-PD-1/PD-L1 antibodies are considered risk factors [31]. In the present study, the eGFR value was approximately 40 mL/min in the two cases treated with nivolumab, whereas it was 105 mL/min in the case treated with pembrolizumab, indicating that acute kidney injury occurred regardless of the eGFR value. There was also no difference in the use of PPIs between the irAE group and the non-irAE group. There have been some reports of acute kidney injury from the start of ICI administration to the onset of symptoms varying from 9 to 42 weeks [1]. In a large retrospective study, the median time to onset was 14 weeks, of which 30% occurred between weeks 1 and 5, and approximately 5% occurred after 90 weeks [31]. In the present study, one case each occurred during the third and fifth courses of nivolumab. With respect to pembrolizumab, one case occurred during the fifth course, indicating that the onset was within the median time of previous retrospective studies.

#### 4.2.6. Adrenal Insufficiency

Adrenal sufficiency is rare in irAEs. In the WHO pharmacovigilance database, 50,108 cases of irAEs were reported, of which 451 (0.9%) were the result of adrenal insufficiency (45 confirmed cases, 406 suspected cases) [32]. There was also a report that when anti-CTLA-4 and anti-PD-1 antibodies were combined, the rate was 5.2–7.6% [19]. In the present study, the incidence of adrenal insufficiency was 2.0% (2 of 80 cases), which is slightly higher than that of previous reports. Adrenal insufficiency with nivolumab occurred in 1% of patients, with a median time to onset of 4.3 months (IQR: 15 days to 21 months) [21]. In the present study, one case occurred during the 47th course of nivolumab and one case occurred during the 3rd course of atezolizumab.

### 4.3. Limitations

There are several limitations to this study. First, this was a single-center retrospective observational study with a small number of cases, which did not provide adequate power to assess the risk factor of irAE. Although Yamaguchi et al. reported that pre-existing autoimmune diseases are a risk factor for irAEs in a meta-analysis [33], no such association was observed in this study, which may be due to the small number of samples. Second, minor cases that did not result in a diagnosis of irAEs and cases with irAEs that developed after the observation period were not included, and the development of irAE for each type of cancer was not examined in this study. Third, our sample was focused on only a Japanese population and may not generalize to other patients, including those of different races. Despite these limitations, it describes not only the incidence, timing, and symptoms of irAEs, but also the treatment for irAEs and subsequent cancer treatments, which may help in treatment strategies in actual clinical practice. Further research is required to enhance treatment strategies and patient outcomes in the future.

## 5. Conclusions

In conclusion, our findings suggest a high probability of irAEs occurring early in ICI treatment, with a diverse range of symptoms. This underscores the need for vigilant monitoring and tailored patient management during the initial courses of ICI therapy. At our hospital, we created an “irAE check sheet” and asked patients to check their own symptoms. We believe that patient self-monitoring is essential for the early detection of irAEs.

## Figures and Tables

**Table 1:** Clinical characteristics between the irAE group and non-irAE group.

		irAE		Non-irAE		*p*-Value
Clinical Characteristics		*n* = 27		*n* = 53		
Male gender		21	(77.8)	40	(75.5)	0.819
Age (years) ^a^		68	(65–78)	71	(65–76)	0.963
BMI (kg/m^2^) ^a^		20.9	(17.8–23.8)	20.1	(17.4–22.6)	0.274
History of ILD		3	(11.1)	4	(7.6)	0.683
History of COPD		3	(11.1)	7	(13.2)	>0.999
History of autoimmune disease		1	(3.7)	1	(1.9)	>0.999
PPI ^b^		6	(22.2)	15	(28.3)	0.559
Concomitant use of ipilimumab		4	(14.8)	0	(0)	0.011
Anti-TPO antibody		13	(48.2)	9	(17.0)	0.003
Anti-Tg antibody		11	(40.7)	9	(17.0)	0.020
Laboratory data ^a^						
	WBC (10^3^/µL)	6.6	(5.1–8.6)	6.7	(5.4–9.9)	0.665
	RBC (10^4^/µL)	417	(347–444)	382	(335–428)	0.105
	Hb (g/dL)	12.3	(10.2–14.0)	11.2	(10.0–12.6)	0.100
	PLT (10^4^/µL)	25.3	(19.8–35.4)	25.6	(18.7–35.4)	0.947
	CCr (mL/min)	60.9	(46.5–85.2)	53.5	(41.9–71.5)	0.114
	eGFR (mL/min)	69.6	(50.9–85.0)	63.7	(47.4–76.2)	0.255
	T-Bil (mg/dL)	0.6	(0.5–1.0)	0.6	(0.4–0.8)	0.201
	AST (U/L)	23	(20–28)	22	(18–37)	0.988
	ALT (U/L)	17	(13–22)	16	(10–31)	0.760

ALT, Alanine aminotransferase; AST, aspartate aminotransferase; BMI, body mass index; CCr, creatinine clearance; COPD, chronic obstructive pulmonary disease; eGFR, estimated glomerular filtration rate; ILD, interstitial lung disease; irAE, immune-related adverse event; PLT, platelet; PPI, proton pump inhibitor; RBC, red blood cell; T-Bil, total bilirubin; Tg, thyroglobulin; TPO, thyroid peroxidase; WBC, white blood cell. ^a^: Values are presented as median (interquartile range). ^b^: *n* = 68.

**Table 2:** Grade of irAEs, treatment for irAE, and subsequent cancer therapy for irAE group.

Patient ID ^a^	Age	Gender	ICI	irAE (Organ)	Grade	Hospitalization Due to irAE	Discontinuation of ICI Treatment	Treatment for irAE	Subsequent Cancer Therapy
1	67	M	NIVO	Lung	3	YES	YES	Prednisolone	None
2	72	M	NIVO	Lung	2	NO	YES	Prednisolone	None
3	79	M	NIVO	Lung	2	NO	YES	Prednisolone	Other chemotherapy
4	67	M	NIVO	Skin	3	NO	NO	Prednisolone	Continuation of NIVO
5	77	M	NIVO	Skin	2	NO	NO	Antihistamines	Continuation of NIVO
6	66	M	NIVO	Skin	2	NO	NO	Antihistamines	Continuation of NIVO
7	77	M	NIVO	Thyroid	2	NO	YES	Levothyroxine	None
8	84	M	NIVO	Thyroid	2	NO	YES	Levothyroxine	None
9	28	M	NIVO	Renal	3	YES	YES	Prednisolone	Resuming NIVO
10	82	F	NIVO	Renal	2	YES	YES	Ringer’s acetate solution	None
11	66	M	NIVO	Liver	2	NO	YES	UDCA	Resuming NIVO
12	50	F	NIVO	Adrenal gland	1	NO	NO	Hydrocortisone	Continuation of NIVO
13	81	M	PEMBRO	Lung	3	YES	YES	None	Changed to ATEZO
14	78	M	PEMBRO	Lung	3	YES	YES	Prednisolone	None
15-2	62	F	PEMBRO	Lung	3	YES	YES	Prednisolone	None
16-2	58	M	PEMBRO	Lung	2	YES	YES	Prednisolone	Other chemotherapy
17	76	F	PEMBRO	Lung	2	YES	YES	Prednisolone	None
18	66	M	PEMBRO	Skin	2	NO	NO	Topical steroids	Continuation of PEMBRO
19	81	M	PEMBRO	Skin	2	NO	NO	None	Continuation of PEMBRO
20	67	M	PEMBRO	Skin	2	NO	NO	Antihistamines	Continuation of PEMBRO
21	63	M	PEMBRO	Thyroid	2	NO	NO	Levothyroxine	Continuation of PEMBRO
16-1	58	M	PEMBRO	Thyroid	2	NO	NO	Levothyroxine	Continuation of PEMBRO
15-1	62	F	PEMBRO	Thyroid	1	NO	NO	None	Continuation of PEMBRO
22	68	F	PEMBRO	Renal	2	NO	YES	None	Resuming PEMBRO
23	92	M	PEMBRO	Liver	2	NO	YES	UDCA	None
24	69	M	ATEZO	Lung	3	YES	YES	Prednisolone	None
25	65	F	ATEZO	Lung	2	YES	YES	Prednisolone	Other chemotherapy
26	72	M	ATEZO	Skin	1	NO	YES	Topical steroids	Other chemotherapy
27	62	M	ATEZO	Adrenal gland	1	NO	YES	Hydrocortisone	None

ATEZO, atezolizumab; F, female; ICI, immune-checkpoint inhibitor; irAE, immune-related adverse event; M, male; NIVO, nivolumab; PEMBRO, pembrolizumab; UDCA, ursodeoxycholic acid. ^a^: Patients 15 and 16 both developed two irAEs. These patients first experienced thyroid dysfunction (IDs 15-1 and 16-1) and later developed pneumonitis (IDs 15-2 and 16-2).

## Data Availability

Data are contained within the article.
